# Cognitive tests distinguish biomarker-verified early Alzheimer’s disease from other patients

**DOI:** 10.1186/s12883-026-04742-7

**Published:** 2026-03-06

**Authors:** Franziska Kiene, Annika Notbohm, Mandy Roheger, Thomas Duning, Helmut Hildebrandt

**Affiliations:** 1 Department of Psychology, Carl von Ossietzky Universität Oldenburg, Ammerländer Heerstr. 114-118, Oldenburg, 26129 Germany; 2 Department of Neurology, Klinikum Bremen-Ost, Züricher Str. 40, Bremen, 28325 Germany

**Keywords:** Cerebrospinal fluid, Cognitive functioning, Early diagnosis, Neurodegenerative disease, Neuropsychological assessment, Paired-associate learning

## Abstract

**Background:**

The diagnosis of Alzheimer’s disease (AD) is based on the presence of characteristic biomarkers and the presence of a clinical AD phenotype. Since pathological changes typically begin years before a formal diagnosis, early detection is an important objective in AD research. Literature suggests that especially cognitive tests for assessing relational memory and memory tests requiring discrimination between previously learned and similar novel stimuli, are indicative of early AD-related cognitive impairment.

**Methods:**

288 memory clinic patients were divided into three Mini-Mental State Examination (MMSE) groups: Group A (MMSE 27–30), Group B (MMSE 23–26), Group C (MMSE 18–22). Logistic regression analyses were conducted to evaluate the ability of seven neuropsychological tests, selected for their clinical relevance in diagnosing AD and distinguishing it from differential diagnoses, to predict whether a patient has AD according to their cerebrospinal fluid profile (AD+) or another neurodegenerative disease/ another dementia syndrome (AD-), for the three MMSE groups separately.

**Results:**

The model’s classification accuracy decreased from 83.78% in Group A to 72.92% in Group B and 75.81% in Group C. In Group A, significant predictors included Paired-Associate Learning (PAL), with AD+ patients performing worse; Phonemic Fluency (PhonoWF) and Beck’s Depression Inventory (BDI). PhonoWF and BDI are related to impairments of the AD- group: AD- patients showed a poorer test performance and more depressive symptoms.

**Conclusions:**

Predictive value of neuropsychological tests varies with the stage of cognitive impairment. Integrating relational memory tasks, such as PAL, enhances the utility of neuropsychological assessments in early identification of clinical AD phenotypes.

## Background

Alzheimer’s disease (AD) is a neurodegenerative disease with pathological changes that often begin years before the formal diagnosis. Accordingly, early detection is an important objective in AD research.

The International Working Group of AD recommends that the diagnosis of AD should be *clinical–biological*. Therefore, it requires the presence of a specific clinical AD phenotype as well as biomarker evidence of AD pathology (amyloid-positive and tau-positive), e.g. tested with cerebrospinal fluid (CSF) analysis [1]. The *amnestic syndrome of the hippocampal type* is the typical clinical phenotype associated with AD pathology [2]. Hippocampal type memory deficits comprise deficient free recall, lacking efficiency in the use of cues and high number of intrusions during cognitive tests [2]. However, according to the Braak stages [3], AD-related tau pathology in the hippocampus mainly starts in stage III and IV of the disease [4, 5], but for early AD detection, Braak stages I and II are more relevant, showing pathological biomarker changes mainly in the transentorhinal- (stage I) and entorhinal cortex (EC) (stage II) [4]. Although morphological changes according to the Braak stages are contributing to the clinical symptoms of AD, these pathological changes are not perfectly correlated with the clinical symptoms [6].

The lateral EC is associated with the processing of non-spatial memory, comprising object recognition. It contains neurons that encode objects (object cells) and their associated locations (trace cells). Therefore, pathology in this area could lead to an impaired object-location coding. It has been shown that related to the accumulation of amyloid beta (Aβ) and tau, lateral EC neurons in animals present a reduced firing precision. Object- and trace cells fire with reduced precision, which leads to a poor encoding of objects and therefore object-context memory deficits/ deficits in the recognition of objects in a specific context [7]. Also, other animal studies are showing that lesions in the area of the EC lead to impairments in tasks requiring the flexible use of relational memory (paired-associate learning tasks). Such lesions do not seem to impact memory for individual items [8, 9], which is commonly tested with word list learning paradigms in humans. Trelle et al. [10] assessed CSF AD biomarker in cognitively unimpaired older adults and all participants completed three different memory tasks: standard delayed recall, paired-associate learning (word-picture) and a memory task focusing on the discrimination of learned- (target) versus new (distractor) objects with perceptually similar stimuli. Only the two latter tests were related to phosphorylated tau_181_ (p-tau_181_) [10], showing that commonly used word list learning paradigms alone may not be the optimal choice for the early detection of AD.

These considerations lead to the assumption that standard neuropsychological assessment protocols for early AD detection should be complemented by cognitive tests that assess object recognition ability for similar objects and tests that assess relational memory. In this study, we analysed the ability of seven neuropsychological tests, including German CERAD-NAB Plus subtests (Consortium to Establish a Registry for Alzheimer’s Disease - Neuropsychological Assessment Battery [11–14]), an object recognition test and a paired-associate learning test, to predict whether a patient has AD or another neurodegenerative disease/ another dementia syndrome, according to their biomarker profile, for early and later stages of cognitive impairment, measured by the German version of the MMSE (Mini-Mental State Examination) [15], which is included in the CERAD test battery. The novel contribution of our study lies in the integration of tests assessing object recognition ability and relational memory into a standard neuropsychological assessment protocol used in clinical practice and their diagnostic evaluation in a clinical sample. This is particularly relevant, as the early detection of biomarker-positive AD and its differentiation from other diagnoses in routine clinical settings is crucial for timely interventions, for example, emerging AD treatment with antibodies that are indicated only for individuals with specific biomarker abnormalities.

## Methods

### Patients

We collected data from 331 patients with a cognitive impairment, admitted to the neurological department of our hospital over the last five years (2019 to 2024). Patients with causes of their cognitive impairment that were neither degenerative nor attributable to another dementia syndrome, were not included in the study (e.g. stroke or encephalitis patients). Patients younger than 52 years of age or with a total tau score beyond 1900 pg/ml were excluded, as a total tau level of 1900 pg/ml and above indicates either an acute cause or Creutzfeldt-Jakob disease [16]. Patients with an MMSE score below 18 usually perform on a floor level in neuropsychological tests, which was the reason why they were excluded as well. The final sample consisted of 288 patients.

Patients were classified into AD patients (AD+) and non-AD patients (AD-) according to their CSF p-tau/ Aβ_42_ ratio with a cut-off score of > 0.0867, as presented by Tapiola et al. [17], who conducted post-mortem analyses of the brains for diagnosing AD and defining cut-off values [17]. The AD- group was not a healthy control group, but consisted of patients with other neurodegenerative conditions or another dementia syndrome: dementia syndrome (*n* = 12), dementia with Lewy bodies (DLB) (*n* = 2), frontotemporal dementia (FTD) (*n* = 3), mild cognitive impairment (MCI) (*n* = 52), multiple system atrophy (*n* = 1), no diagnosis (*n* = 2), normal pressure hydrocephalus (*n* = 14), Parkinson’s disease (PD) (*n* = 8), progressive supranuclear palsy (*n* = 3), semantic dementia (*n* = 2), (suspected) AD (*n* = 19), vascular dementia (VD) (*n* = 4).^1^

The MMSE cognitive screening tool was used to classify the patients into three subgroups, according to the stage of their cognitive impairment. Higher MMSE scores are indicating a better test performance and the maximum score is 30 [15]. We acknowledge that the MMSE is a widely used global screening tool, however, it cannot replace a comprehensive clinical assessment to get a final diagnosis [15]. The first group (Group A) consisted of patients who scored 27–30 in the MMSE (*n* = 86, Table 1). Patients in Group B scored 23–26 in the MMSE (*n* = 118, Table 1). Group C consisted of patients with a MMSE score of 18–22 (*n* = 84, Table 1). Literature is indicating that older patients, who score below 27 on the MMSE, which is the cut-off score to divide Group A and B of the present study, are at elevated risk of cognitive dysfunction and dementia [18]. MMSE scores of 23/24 are often used as cut-off for classifying mild dementia and a cut-off score of ≤ 17 is recommended to classify moderate dementia [19].

**Table 1:** Sample characteristics

Variable	Group A: MMSE 27–30 *n* = 86	Group B: MMSE 23–26 *n* = 118	Group C: MMSE 18–22 *n* = 84	Global ANOVA *p* value
	Mean (SD)	Mean (SD)	Mean (SD)	
Sex (m/f)	47/39	61/57	39/45	/
Age	69.54 (8.10) ***	71.90 (7.75)	74.11 (7.15) ***	**< 0.001**
Years of education	13.84 (2.41) *	13.68 (2.64)	12.86 (2.81) *	**0.032**
MMSE	28.00 (1.05) ***	24.61 (1.05) ***	20.48 (1.36) ***	**< 0.001**
Total tau	465.00 (289.52) *	555.39 (350.85)	598.83 (394.49) *	**0.038**
P-tau_181_	63.75 (48.38)	73.97 (48.58)	78.84 (58.23)	0.148
Aβ_42_	754.33 (378.60) ***	574.40 (289.14) ***	525.24 (267.95) ***	**< 0.001**

Data presented as *n* or *mean* (*SD*). *p* < .05 *; *p* < .01 **; *p* < .001 ***; Significant values are in bold

Significant group differences in Aβ_42_ were found between Group A and B, and between Group A and C

Abbreviations: *Aβ*_42_ amyloid beta 42, *f* female, *m* male, *MMSE* Mini-Mental State Examination, *p-tau*_181_ phosphorylated tau_181,_*SD* standard deviation

Therefore, we chose this cut-off to delimit the third group at the lower end. Characteristics of the sample can be found in Table 1.

The retrospective re-evaluation project was approved by the ethics committee of the medical board of Bremen, number 905/ 2024, and was performed in accordance with the ethical standards of the Declaration of Helsinki.

### Neurological examination

The standard diagnostic examination protocol included medical history assessment, physical and neurological examination, laboratory testing, brain imaging and CSF analysis. The extensive blood sample analysis included blood count, erythrocyte sedimentation rate, electrolytes (sodium, potassium, chloride), creatine, urea, transaminases, blood glucose, TSH, C-reactive protein, vitamin B 12, folic acid. Optional further blood analyses included B-vitamins, TPPA, immunological parameter, HIV, and copper metabolism. Approximately 5 ml CSF was collected. CSF samples were analysed for cell count, total protein, lactate, glucose, IgG, IgA, IgM, borrelioses antibodies.

CSF total tau, p-tau_181_ and Aβ_42_ were determined quantitatively using Fujirebio LUMIPULSE G assays per manufacturer’s instructions. All analyses were performed commercial at the LADR GmbH MVZ Bremen.

### Neuropsychological examination

**Fig. 1:**
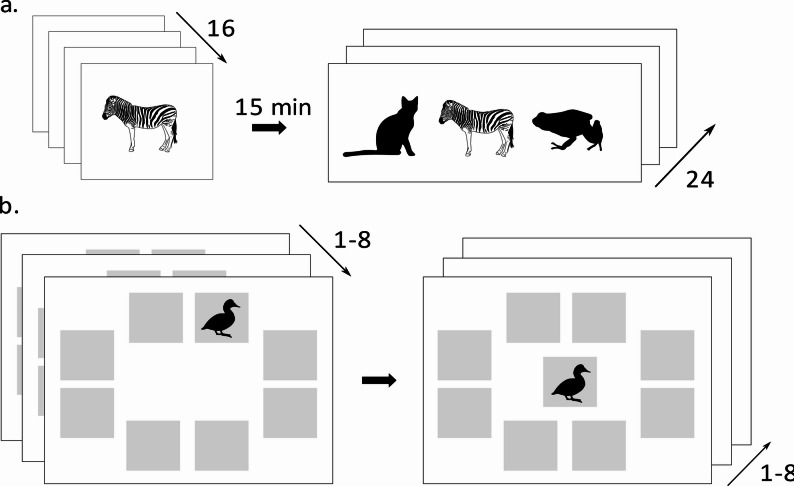
**a** Picture Recognition Test. 16 pictures are presented during learning phase. The recognition phase, where three pictures are shown, takes place after 15 min. In 24 trials, participants have to decide, whether one of three pictures was presented before during learning phase and if so, which one. Omissions and false positives are counted. **b** Paired-Associate Learning Test (PAL). A drawing presented at the centre of a computer screen has to be assigned to the location (grey fields) where it was previously presented. The test consists of different difficulty levels with an increasing number of drawings (1-8), that have to be assigned to the correct locations. PAL tests object-location memory

All patients underwent a neuropsychological examination, including the German CERAD-NAB Plus version [11–13]. In addition, the Digit Span Forward and -Backward Task from the German version of the Wechsler Memory Scale [20] was conducted and the Beck’s Depression Inventory (BDI) [21] was used to assess depressive symptoms. Additionally, a picture recognition test as described in Hildebrandt et al. [22] was conducted on the computer (Fig. 1a) [22]: 16 pictures are presented to the patient during learning phase. In the recognition phase, the patient has to decide whether one of three pictures was presented before during learning phase and if so, which one. Also, a self-developed object-location memory test, the Paired-Associate Learning Test (PAL), was conducted on the computer (Fig. 1b). Objects are presented to the patient at one of eight different locations on the screen, starting with one object at one location (= one pair of object and location) at the first difficulty level. Patients have to memorize the object and its correct location. The test consists of five difficulty levels with one, two, four, six or eight objects at different locations. An object is shown for three seconds at its location, followed immediately by the next object at another location. After all objects of the respective difficulty level were presented at their location, they are presented again at the centre of the screen one after another in a random order. The patient has to point to the location where the object was initially presented. Up to three trials per difficulty level are conducted. A level is completed if the correct location of all objects of this level is identified. If a patient is not able to identify the correct location of all objects of one level within three trials, the test stops and the last completed level is used for the performance scoring. Objects are black and white drawings of items that occur with a moderate frequency in everyday life. The object locations are marked by rectangles at fixed locations. 

The whole assessment, including anamnesis, lasted about 1.5 h and was carried out in one diagnostic session.

### Statistics

Significance level for all analyses was set at *p* < .05 if not stated otherwise. Analyses were performed using IBM SPSS Statistics Version 30.0.0.0. In a first step, we conducted an ANOVA (Table 1) with post-hoc tests using Tukey-HSD to compare characteristics of the patients of different MMSE groups.

As explained above, we applied the cut-off value presented by Tapiola et al. (2009) [17] to divide our sample in AD+ and AD- patients according to their CSF biomarker profile. To investigate whether neuropsychological tests are able to predict group membership AD+ vs. AD-, we calculated a logistic regression for each of the three MMSE groups separately (Group A: MMSE 27–30, Group B: MMSE 23–26, Group C: MMSE 18–22), using the two groups of interest (AD+ vs. AD-) as dichotomous outcome variable. Simulation studies indicate that the general rule of thumb of 10 events per predictor variable (EPV) in logistic models is too conservative. Problems are uncommon also with 5 EPV [23]. Based on the sample size of *n* = 84 of the smallest group (MMSE Group C), we did not include more than seven predictors. Seven neuropsychological tests were defined in advance as predictors for the logistic regression analyses, based on their clinical relevance for diagnosing AD and distinguishing it from differential diagnoses. Z-scores adjusted for age-, sex-, and education were used wherever possible. The same seven neuropsychological tests were employed for each logistic regression:

1. Test scores of the ‘Paired-Associate Learning Test’ (*PAL*). Higher scores (more correct remembered objects at correct locations) are indicating a better test performance.
2. False positives of the ‘Picture Recognition Test’ (*RecFP*). Higher scores (more falsely recognized pictures) are indicating a worse test performance. We included the two latter tests due to the reasons explained in the introduction.
3. Z-scores of the CERAD-NAB coefficient ‘Word List Savings’ (*WLsavings*). Higher scores are indicating a better test performance [24].
4. Z-scores of the CERAD-NAB coefficient ‘Figure Savings’ (*Figsavings*). Higher scores are indicating a better test performance. The two latter tests were included as literature is indicating that verbal memory and savings- and visual memory neuropsychological performance is starting to deteriorate measurably years prior to a MCI due to AD diagnosis [24].
5. Correct negatives of the CERAD-NAB subtest ‘Word List Recognition’ (*WLReco*). Higher scores (more unknown words correctly identified as unknown) are indicating a better test performance. This subtest was included, as AD patients tend to make false positive errors, resulting in less correct negatives.
6. Z-scores of the CERAD-NAB Plus test ‘Phonemic Fluency (S-Words)’ (*PhonoWF*). Higher scores are indicating a better test performance [24]. We included this subtest due to its relevance for a differential diagnosis, as phonemic fluency is not primarily impaired in early AD but rather in other forms of dementia [25].
7. Test scores of the ‘Beck’s Depression Inventory’ (*BDI*). Higher scores are indicating more depressive symptoms [21]. This test was also included due to its relevance for a differential diagnosis.

Mean test performance and standard deviations were calculated and compared with a t-test for the seven neuropsychological tests (predictors in the logistic regression analyses) for AD+ and AD- patients (Table 2). Results of the logistic regressions are presented in Table 3 as odds ratios (OR), which quantify the association between each independent variable and the dependent variable. Multicollinearity among independent variables was assessed and the linearity of continuous predictors with the logit was tested. No significant multicollinearity was detected. Model fit was evaluated using the model chi-square statistic. With a *p* value < 0.05 the overall model with the seven predictors is predicting whether a patient has AD or not significantly better than a model without the predictors [26]. Also, model fit was evaluated using Hosmer-Lemeshow goodness-of-fit test, with a *p* value > 0.05 indicating an adequate fit. Nagelkerke R-squared value was calculated as effect size. In the context of the logistic regression analyses, predicted probabilities to belong to the AD+ group were calculated for each patient. A probability of 0.5 is the threshold to classify a case as either AD+ or AD-. Patients with a predicted probability of < 0.5 are classified by the model as belonging to the AD- group. However, due to different group sizes in some MMSE groups, we have calculated a receiver operating characteristic (ROC) analysis with the predicted probabilities of the logistic regression analyses and have determined the optimal probability threshold to classify patients as either AD+ or AD-, based on the maximum Youden Index (J) [27] (J = sensitivity + specificity − 1).

**Table 2:** Neuropsychological test performance of AD+ and AD- patients

Neuropsychological test	Group A: MMSE 27–30		Group B: MMSE 23–26		Group C: MMSE 18–22	
	AD- *n* = 50	AD+ *n* = 36	AD- *n* = 45	AD+ *n* = 73	AD- *n* = 27	AD+ *n* = 57
	Mean (SD)	Mean (SD)	Mean (SD)	Mean (SD)	Mean (SD)	Mean (SD)
PAL	5.48** (2.24)	4.12** (2.09)	3.19 (1.35)	3.24 (1.86)	2.96 (1.60)	2.49 (1.29)
RecFP	0.56* (0.84)	2.06* (3.59)	1.55*** (1.69)	3.83*** (4.15)	2.24*** (2.82)	5.13*** (4.13)
WLsavings	-0.54** (1.71)	-1.51** (1.40)	-1.50* (1.90)	-2.35* (1.69)	-1.85* (1.57)	-2.65* (1.66)
Figsavings	-0.98 (1.14)	-1.27 (1.20)	-1.74 (1.01)	-2.11 (0.95)	-1.94 (1.14)	-2.23 (0.89)
WLReco	9.94* (0.24)	9.61* (0.77)	9.78*** (0.60)	9.03*** (1.28)	8.37 (2.11)	8.36 (2.17)
PhonoWF	-0.82*** (1.18)	0.14*** (1.01)	-1.16** (1.31)	-0.45** (0.99)	-1.22 (1.32)	-1.10 (1.24)
BDI	11.18 (9.04)	8.17 (6.91)	10.07 (7.95)	8.21 (6.22)	10.00 (8.37)	7.60 (6.37)

Data presented as *mean* (*SD*). Welch’s t-test, *p* < .05 *; *p* < .01 **; *p* < .001 ***. AD- means non-AD patients, AD+ means AD patients according to their CSF biomarker status

Abbreviations: *AD* Alzheimer’s disease, *BDI* Beck’s Depression Inventory, *CSF* cerebrospinal fluid, *Figsavings* Figure Savings (z-scores), *MMSE* Mini-Mental State Examination, *PAL* Paired-Associate Learning, *PhonoWF* Phonemic Word Fluency (z-scores), *RecFP* Picture Recognition false positives, *SD* standard deviation, *WLReco* Word List Recognition, *WLsavings* Word List Savings (z-scores)

**Table 3:** Results of the logistic regression analyses for the three MMSE groups

MMSE Group	Predictor	OR (95% CI)	*p* value
A: MMSE 27–30	PAL	0.58 (0.40–0.86)	**0.006**
	RecFP	1.35 (0.78–2.34)	0.282
	WLsavings	0.64 (0.38–1.10)	0.105
	Figsavings	1.18 (0.64–2.18)	0.588
	WLReco	0.27 (0.06–1.09)	0.066
	PhonoWF	2.75 (1.34–5.64)	**0.006**
	BDI	0.87 (0.77–0.99)	**0.035**
	**Model fit**	*n/ R²/ Percentage* classification threshold 0.50	*n/ R²/ Percentage* classification threshold 0.46
	*n*	74 (32 AD+)	74 (32 AD+)
	Total *R²*	0.60	0.60
	Sensitivity	81.25	90.63
	Specificity	85.71	83.33
	Accuracy	83.78	86.49
B: MMSE 23–26	PAL	1.19 (0.86–1.65)	0.285
	RecFP	1.33 (1.01–1.74)	**0.039**
	WLsavings	0.98 (0.72–1.35)	0.918
	Figsavings	0.71 (0.41–1.25)	0.238
	WLReco	0.34 (0.13–0.88)	**0.025**
	PhonoWF	1.87 (1.17-3.00)	**0.009**
	BDI	0.95 (0.88–1.03)	0.229
	**Model fit**	*n/ R²/ Percentage* classification threshold 0.50	*n/ R²/* *Percentage * classification threshold 0.56
	*n*	96 (57 AD+)	96 (57 AD+)
	Total *R²*	0.43	0.43
	Sensitivity	78.95	75.44
	Specificity	64.10	74.36
	Accuracy	72.92	75.00
C: MMSE 18–22	PAL	0.62 (0.39-1.00)	0.052
	RecFP	1.25 (0.99–1.56)	0.055
	WLsavings	0.72 (0.43–1.18)	0.192
	Figsavings	0.81 (0.40–1.65)	0.560
	WLReco	1.21 (0.87–1.69)	0.262
	PhonoWF	1.39 (0.78–2.50)	0.267
	BDI	1.00 (0.92–1.09)	0.962
	**Model fit**	*n/ R²/ Percentage* classification threshold 0.50	*n/ R²/ Percentage * classification threshold 0.63
	*n*	62 (42 AD+)	62 (42 AD+)
	Total *R²*	0.34	0.34
	Sensitivity	92.86	76.19
	Specificity	40.00	80.00
	Accuracy	75.81	77.42

Outcome variable was group membership AD+ or AD-. AD- means non-AD patients, AD+ means AD patients according to their CSF biomarker status. *R*^*2*^ = Nagelkerke R-squared value. The classification threshold is the predicted probability which the model uses as a threshold to classify a case as either AD+ or AD-. Classification thresholds on the right side were calculated based on ROC analyses of predicted probabilities of the logistic regression analyses. Youden Index was used to determine the optimal threshold; Significant values are in bold

Abbreviations: *AD* Alzheimer’s disease, *BDI* Beck’s Depression Inventory, *CI* Confidence Interval, *CSF* cerebrospinal fluid, *Figsavings* Figure Savings, *MMSE* Mini-Mental State Examination, *OR* odds ratio, *PAL* Paired-Associate Learning, *PhonoWF* Phonemic Word Fluency, *RecFP* Picture Recognition false positives, *ROC* receiver operating characteristic, *WLReco* Word List Recognition, *WLsavings* Word List Savings

As we were especially interested in early diagnostics, we calculated optimal cut-off values to identify Group A patients with AD+ for the significant neuropsychological tests of the logistic regression (Table 4). To determine optimal cut-off values, the maximum Youden Index (J) [27] of ROC curves was used [28]. Cut-off values were derived from separate ROC analyses, whereby each analysis was based on a single neuropsychological test.

**Table 4:** Optimal cut-off values to classify group A patients into AD+ and AD-

Neuropsychological test	Optimal cut-off according to J (J value)	AUC (95% CI)	Sensitivity	Specificity
PAL	≤ 7.00 (0.24)	0.67 (0.55 − 0.79)	0.88	0.36
PhonoWF	≥ -0.55 (0.37)	0.72 (0.61 − 0.83)	0.74	0.63
BDI	≤ 9.50 (0.19)	0.60 (0.47 − 0.72)	0.78	0.41

Group A: MMSE 27–30. AD+ if ≥ or ≤ the stated cut-off value. AD- means non-AD patients, AD+ means AD patients according to their CSF biomarker status. Cut-off values were derived from separate ROC analyses, whereby each analysis was based on a single neuropsychological test

Abbreviations: *AD* Alzheimer’s disease, *AUC* Area under the (ROC) curve, *BDI* Beck’s Depression Inventory, *CI* confidence interval, *CSF* cerebrospinal fluid, *J* Youden Index, *MMSE* Mini-Mental State Examination, *PAL* Paired-Associate Learning, *PhonoWF* Phonemic Word Fluency (z-score), *ROC* receiver operating characteristic

## Results

### Sample characteristics

Patient characteristics for the three MMSE groups are presented in Table 1. Results of the ANOVA with post-hoc tests show that Group A patients were significantly younger (MD: -4.57, *p* < .001) and better educated (MD: 0.98, *p* < .05) than Group C patients. Also, Group A patients had a lower total tau than Group C patients (MD: -133.83, *p* < .05). Group A patients displayed significantly higher Aβ_42_ values than Group B patients (MD: 179.93, *p* < .001) and Group C patients (MD: 229.09, *p* < .001). There were no group differences regarding p-tau_181_ levels.

### Predicting group membership AD+ or AD- with neuropsychological tests

Logistic regression analyses results for the three MMSE groups to investigate whether neuropsychological tests are able to predict group membership AD+ or AD- are presented in Table 3 and the mean test performance of patients in the seven neuropsychological tests that were used as predictors are presented in Table 2.

The model’s overall classification accuracy decreased from 83.78% in MMSE Group A to 72.92% in MMSE Group B and 75.81% in MMSE Group C. The effect size also decreased from a large effect in Group A to a medium effect in Group B and only a small effect in Group C. In MMSE Group A and B, three of the seven neuropsychological tests yielded significant regression coefficients, whereas in MMSE Group C no neuropsychological test yielded a significant result.

#### MMSE Group A

The model was statistically significant (*χ²* (7) = 43.40, *p* < .001) and showed a Nagelkerke *R²* of 0.60, indicating a large effect [29]. Hosmer-Lemeshow test was not significant, indicating a good model fit. Predictors with significant regression coefficients were Paired-Associate Learning PAL (*OR* = 0.58, 95% *CI*: 0.40–0.86, *p* < .01), Phonemic Fluency PhonoWF (*OR* = 2.75, 95% *CI*: 1.34–5.64, *p* < .01) and Beck’s Depression Inventory BDI (*OR* = 0.87, 95% *CI*: 0.77–0.99, *p* < .05). This means that with an increasing test performance in the Paired-Associate Learning PAL (more correct remembered objects at correct locations), the odds to be AD+ decrease. On average, the AD- group achieved 5.48 (*SD* = 2.24) scores and the AD+ group only 4.12 (*SD* = 2.09) scores in the PAL. With an increasing Beck’s Depression Inventory BDI score (more depressive symptoms), the odds to be AD+ also decrease. The AD- group scored on average 11.18 (*SD* = 9.04), whereas the AD+ group only scored 8.17 (*SD* = 6.91) in the BDI. Contrary to that, with increasing z-scores of Phonemic Fluency PhonoWF (more correct words in one minute), the odds to be AD+ increase. Mean z-score of the AD- group was −0.82 (*SD* = 1.18) and of the AD+ group 0.14 (*SD* = 1.01), showing that patients with AD demonstrated a better performance in this test.

#### MMSE Group B 

Again, the model was statistically significant (*χ²* (7) = 36.45, *p* < .001) and Nagelkerke *R²* showed a medium effect size of the model with an *R²* of 0.43 [29]. Hosmer-Lemeshow test was not significant, indicating a good model fit. The model showed three significant neuropsychological tests for predicting group membership: Picture Recognition false positives RecFP (*OR* = 1.33, 95% *CI*: 1.01–1.74, *p* < .05) indicating that with increasing false positives in the Picture Recognition Test, the odds of being AD+ increase. On average, the AD- group showed only 1.55 (*SD* = 1.69) false positives, whereas the AD+ group showed 3.83 (*SD* = 4.15) false positives; Word List Recognition WLReco (*OR* = 0.34, 95% *CI*: 0.13–0.88, *p* < .05) indicating that with increasing correct negatives of the CERAD Word List Recognition task (more unknown words correctly identified as unknown), the odds of being AD+ decrease. The AD- group showed on average 9.78 (*SD* = 0.60) correct negatives and the AD+ group 9.03 (*SD* = 1.28); Phonemic Fluency PhonoWF (*OR* = 1.87, 95% *CI*: 1.17-3.00, *p* < .01), indicating that with increasing z-scores of PhonoWF (more correct words in one minute), the odds to be AD+ increase. Mean z-score of the AD- group was −1.16 (*SD* = 1.31) and of the AD+ group −0.45 (*SD* = 0.99), showing that again patients with AD demonstrated a better test performance in this test.

#### MMSE Group C

The model was statistically significant (*χ²* (7) = 17.52, *p* < .05). Nagelkerke *R²* of this model was 0.34, indicating a small effect [29]. No neuropsychological tests yielded significant regression coefficients in predicting the group membership AD+ or AD-.

Since we were particularly interested in the neuropsychological tests relevant for early diagnosis of AD, we calculated optimal cut-off values to classify MMSE Group A patients into AD+ or AD- based on the Youden index (J) of ROC-curves, for the significant predictors of the logistic regression. The results are displayed in Table 4.

## Discussion

We analysed the ability of seven neuropsychological tests, selected based on their clinical relevance for diagnosing AD and distinguishing it from differential diagnoses, to predict whether patients with cognitive impairments have AD according to their biomarker profile or another neurodegenerative condition/ another dementia syndrome. We conducted the analyses for early and later stages of cognitive impairment and focused on the question whether additional neuropsychological tests, especially a paired-associate learning- and a picture recognition test, beyond the classical word list paradigms, are valuable for early AD diagnosis.

Logistic regression analyses indicated a large effect (*R²* > 0.5) of the model in MMSE Group A (MMSE 27–30) and Paired-associate Learning (PAL), CERAD-NAB Plus Phonemic Fluency (PhonoWF) and the BDI were significant predictors of group membership AD+ vs. AD-. The model with three significant predictors yielded a sensitivity of 81.25% and a specificity of 85.71%.

Our results are consistent with the findings of Trelle et al. [10], who conducted a PAL version in cognitively unimpaired older adults and found significant associations between CSF biomarker of preclinical AD (Aβ_42_/Aβ_40_, p-tau_181_) and the test performance in this associative memory task with word–picture pairs (effect of Aβ_42_/Aβ_40_ was mediated by p-tau_181_ increase) [10]. In our study, AD- patients in Group A achieved on average 5.48 (*SD* = 2.24) scores and AD+ patients only 4.12 (*SD* = 2.09) scores in the PAL. As described above, Braak stages I and II with pathological biomarker changes mainly in the transentorhinal- and EC [4] are relevant for the early detection of AD. Lesions in the area of the EC might lead to impairments in relational memory tasks such as the PAL, giving a possible explanation for the diagnostic value of this task in Group A. Several other studies also demonstrated that paired-associate learning tests are a sensitive tool in an early stage of AD [25, 30, 31], however most of them focused on clinical and not CSF-based diagnosis of AD. Hicks et al. [32], analysed the ability of a comparable PAL task to differentiate between AD (*n* = 25) and healthy controls (*n* = 22) with ROC analyses. Total errors of the PAL yielded 92% sensitivity and 86% specificity [32]. Although the sample size was relatively small and unlike in our study, the comparison was between AD patients and healthy controls, the results nonetheless highlight the diagnostic value of a PAL task in the detection of AD.

Interestingly, the two other significant predictors in Group A were related to impairments of the AD- group, which consisted of patients with other neurodegenerative conditions or another dementia syndrome, like PD, FTD, VD or DLB. In both measurements, depressive symptoms measured with BDI and Phonemic Fluency (PhonoWF), did individuals of the AD- group show a worse test performance/ more depressive symptoms compared to individuals of the AD+ group. Our findings correspond to the results of Wiels et al. [33], who found that depressive symptoms were associated with a lower likelihood of pathological amyloid alterations in individuals with MCI and therefore suggested that alternative mechanisms beyond amyloid accumulation may underlie depressive symptoms in older adults [33]. There are also studies showing slightly different results than the ones from the present study, Crump et al. [34] found that individuals diagnosed with AD or all-cause dementia showed an elevated risk of major depression, whereby risks were highest within the first year after the diagnosis, compared to population-based controls. Yet, the risk was highest among individuals aged ≥ 85 years at diagnosis [34], and the patients of Group A in our study were on average 69.54 years old. A meta-analysis found that although depression is typical among dementia patients, the type of dementia has an influence on the prevalence rates. The prevalence of major depressive disorder was higher in patients with vascular dementia (24.7%) compared to AD (14.8%) [35]. Also, a meta-analysis found a significant higher risk of subsequent PD in patients with prodromal depression, compared to healthy controls. Although future research is needed to investigate whether depression can be seen as an early prodromal symptom or as a risk factor for PD [36], these findings may give a possible explanation, why in MMSE Group A more depressive symptoms were associated with the AD- group, including PD patients. Further research on the predictive value of the BDI in this context would be valuable.

In the early stages of cognitive impairment, individuals of the AD+ group performed better in phonemic fluency tasks, then the AD- group. Existing literature is showing that phonemic fluency is not primarily impaired in early AD but rather in other forms of dementia [25], which were part of the AD- group. Individuals with mild AD have significant greater impairments in semantic category fluency- then in letter fluency tests [37]. Therefore, it seems plausible that in our study, patients in the AD+ group performed better on phonemic/ letter fluency tasks compared to individuals with other diagnoses. This observation applies to the comparison between AD patients and those with different clinical diagnoses; cognitively healthy individuals would likely achieve higher scores in phonemic fluency tasks than AD patients. When comparing neuropsychological profiles of patients with early AD and patients with DLB, DLB patients show lower scores on attention-, visuospatial- and executive-, including phonemic fluency tests, than AD patients, but better performance in memory tests [38]. Delgado-Álvarez et al. [39] conducted neuropsychological assessments with patients with behavioural variant Frontotemporal Dementia (bvFTD) and AD. Statistically significant differences and the largest effect sizes between groups were observed in semantic and phonemic fluency with lower scores in bvFTD than AD, highlighting the diagnostic value of these tasks for the differential diagnosis [39]. Another study found that patients with normal pressure hydrocephalus, a condition with some clinical overlap with other dementia syndromes, showed an eye movement dysfunction, which was correlated with deficits in phonemic fluency and executive functions but not with memory [40]. Moreover, a systematic review concerning the neuropsychological differential diagnosis of AD and VD showed that AD patients outperformed VD patients in phonemic fluency tasks [41]. This is in line with our results (AD-: *M* = -0.82, *SD* = 1.18; AD+: *M* = 0.14, *SD* = 1.01). On the other hand, individuals with VD outperformed individuals with AD in verbal and visual delayed recall. Patients with AD also performed worse than patients with VD in recognition memory tasks [41]. This is reflected in our results as well: Although the recognition memory task of our study, the Picture Recognition Test (RecFP), only was a significant predictor of group membership AD+ vs. AD- in Group B (MMSE 23–26), did AD patients show a worse test performance than patients with other neurodegenerative conditions or another dementia syndrome in all three MMSE groups. Although the lateral EC, which is affected in early Braak stages and which is associated with the encoding of objects and lesions might lead to deficits in the recognition of objects [7], the RecFP to test recognition ability, was not a significant predictor in Group A. It could be that the RecFP was too easy for Group A patients. In the study of Trelle et al. [10], where a picture recognition task was able to detect CSF biomarker variance related to preclinical AD, task difficulty was varied by using perceptually similar pictures, ranging from high to low similarity, and novel pictures [10]. This was not the case in the RecFP.

Cut-off values of the significant tests identified in Group A may offer a practical aid in the assessment of patients in early stages of cognitive impairment, although the identification of clinical AD phenotypes is based on a comprehensive neuropsychological assessment and not on single test scores. The values were derived from separate ROC analyses, each based on a single neuropsychological test, and represent the optimal cut-off points if diagnostic classification of AD+ vs. AD- relied solely on that respective test. Therefore, the respective J-values and AUCs are relatively low. The cut-offs should be interpreted as guidance rather than definitive diagnostic thresholds. For example, in early diagnostics, a BDI score of > 9.50 points more towards another differential diagnosis, rather than AD+. Sensitivity and specificity values for the respective cut-offs presented in Table 4 were determined using the Youden Index. Depending on the relative costs of misclassifying an individual as AD+ when they are not biomarker-positive, vs. failing to identify someone who actually is biomarker-positive, the cut-off values can be adjusted. The cut-offs in Table 4 yielded higher sensitivity, indicating that in this case, the tests are more effective in detecting AD biomarker-positive individuals than in classifying those without the biomarkers but different conditions (in an early stage of cognitive impairment). This appears reasonable for an initial neuropsychological testing, where it is crucial to avoid overlooking individuals who may benefit from further diagnostic procedures.

CERAD-NAB memory subtests did not contribute significantly to the prediction of group membership in MMSE Group A. One explanation for this might be that they measure memory for individual items (item specific memory) and this may not be impaired in early AD stages, as explained in the introduction.

Similar to the RecFP, correct negatives of the CERAD-NAB subtest Word List Recognition (WLReco) were also a significant predictor of group membership in MMSE Group B but not in MMSE Group A. The AD- group showed more correct negatives (*M* = 9.78, *SD* = 0.60) than the AD+ group (*M* = 9.03, *SD* = 1.28). Previous literature is indicating that especially false positives (more false positive errors are resulting in less correct negatives) rather than omissions of correct items are of importance in neuropsychological assessments of patients suspected for AD [22, 25]. A modified version of RecFP and WLReco that increases the difficulty of distinguishing between correct (learned) and incorrect (novel) items may enhance their ability to differentiate patients in Group A.

Logistic regression analyses showed a reduced classification accuracy in Group B (MMSE 23–26) and in Group C (MMSE 18–22), compared to Group A (MMSE 27–30). One possible explanation for this drop might be that over time AD and its differential diagnoses tend to result in a more generalized cognitive impairment. While in early stages, impairments in particular memory tests (e.g. paired-associate learning tests) may be relatively specific to AD+ patients, these distinctions become less pronounced as the diseases progress. With advanced stages of neurodegenerative and dementia-related diseases, memory impairments become prevalent across conditions and therefore reducing the accuracy of memory assessments in differentiating AD+ and AD- patients. Also, for phonemic fluency, the difference in mean performance between the two groups AD+ vs. AD- decreased from 0.96 *SD* in Group A to 0.12 *SD* in Group C (Table 2). Neuropsychological testing could be improved by incorporating a test that shows only minimal decline in AD+ patients over the course of the disease. This requires further research. Furthermore, it should be considered that ceiling effects may be particularly relevant in the context of early diagnostics, as patients with early impairments might achieve scores near the upper limits of neuropsychological tests. Conversely, in later stages of cognitive impairment, floor effects may emerge, as certain tasks become too challenging for patients.

According to several studies, the careful preselection of a restricted number of candidate predictors, guided by subject expertise, is more important for reducing bias than the events per predictor variable [42, 43]. Nevertheless, the sample size of MMSE Group C is relatively small, which may reduce the stability of the logistic regression estimates and may increase the risk of a type II error. In this context, it is recommended to also consider the confidence intervals [23]. Still, it remains possible that we were unable to detect a possible existing effect due to the limited sample size of this group.

This study also has limitations. It is a retrospective analysis based on data from a single site. Replication in a prospective study involving patients from multiple sites is necessary to enhance generalizability. Moreover, the PAL test for paired-associate learning used to assess relational memory, an ability often impaired in early AD, is a self-developed instrument and the scores are not adjusted for age-, sex-, and education, which constrains its generalizability. Yet, it is a visual PAL and therefore language-independent. Although it differentiated between AD+ and AD- patients of Group A (MMSE 27–30), further validation and the development of normative data is required. Future external validation should include administration in prospective studies with demographically diverse samples, as well as in samples with cognitively healthy controls and patients with different neurocognitive disorders and the comparison with other existing relational memory tasks. Independent replication in multi-centre cohorts to confirm its reliability and diagnostic utility is necessary.

The composition of the AD- group, which included patients with other neurodegenerative and dementia-related diseases, can be seen as a limitation due to its heterogeneity. These group encompasses a wide spectrum of pathologies, each with distinct cognitive phenotypes. This heterogeneity complicates the interpretation of impairments, as the observed neuropsychological pattern is not determined by a unitary condition. In the present study, however, the aim was not to explicitly diagnose all neurodegenerative or dementia-related diseases, but rather to distinguish biomarker-positive AD from other conditions through cognitive testing, resulting in a binary classification of AD+ vs. AD-. The composition of the AD- group can also be considered a strength, as this reflects the clinical setting. Individuals attending memory clinics are usually not healthy controls. The present study is based on clinical routine practice, enhancing its ecological validity. Identifying cognitive tests that can detect AD patients within a heterogeneous, initially undefined patient group referred for neuropsychological assessment, rather than merely distinguishing them from healthy controls or one other defined patient group like PD, is highly valuable.

## Conclusions

Our study offers two relevant information for the neuropsychological assessment of patients with suspected AD: (1) Predictive value of neuropsychological tests is dependent on the stage of cognitive impairment, highlighting the importance of selecting specific neuropsychological tests based on the stage of cognitive impairment. (2) Integrating a test for assessing relational memory, such as a paired-associate learning test (e.g. with picture-location), enhances the utility of neuropsychological assessments in identifying early clinical AD phenotypes.

## Data Availability

The datasets used and/or analyzed during the current study are available from the authors on reasonable request.

## Abbreviations

Aβ_42_: amyloid β_42_
AD: Alzheimer’s disease
AUC: Area under the curve
BDI: Beck’s Depression Inventory
CERAD-NAB: Consortium to Establish a Registry for Alzheimer’s Disease Neuropsychological Assessment Battery
CI: confidence interval
CSF: cerebrospinal fluid
EC: entorhinal cortex
EPV: events per predictor variable
Figsavings: Figure Savings
FTD: frontotemporal dementia
J: Youden Index
DLB: dementia with Lewy bodies
MCI: Mild cognitive impairment
MD: mean difference
MMSE: Mini-Mental State Examination
OR: odds ratio
PAL: Paired-associate Learning
PD: Parkinson’s disease
PhonoWF: Phonemic Fluency
p-tau_181_: phosphorylated tau_181_
RecFP: false positives of the Picture Recognition Test
ROC: receiver operating characteristic
SD: standard deviation
VD: vascular dementia
WLReco: Word List Recognition
WLsavings: Word List Savings

## Footnotes

1 MCI can occur due to various underlying causes. In order to evaluate the prognostic value of neuropsychological tests, a clear distinction between the AD + and AD- group was required, based on non-neuropsychological parameters. Accordingly, we chose the biomarker criterion described above. Thus, some individuals may be clinically suspected of having AD, but do not display the respective biomarker profile, mentioned above.
